# The predictive value of writing disorders in patients with Alzheimer's disease

**DOI:** 10.1002/alz70857_097348

**Published:** 2025-12-24

**Authors:** Viacheslav Viktorovich Sushko, Viktor Vasilievich Sushko

**Affiliations:** ^1^ National University, Odessa, Ukraine; ^2^ Odessa National Maritime University, Odessa, Ukraine

## Abstract

**Background:**

Early‐stage symptoms of Alzheimer's progress slowly, making it challenging to assess the patient's condition prognostically. Alzheimer's disease affects fine motor control even in its initial stages. Analyzing handwriting dynamics may serve as an effective, inexpensive, and non‐invasive tool for predicting the course of Alzheimer's disease.

**Method:**

Over a two‐year period, we observed 12 women aged 65 to 75 diagnosed with Alzheimer's disease. They underwent a graphological assessment annually, following a protocol we developed consisting of 15 tasks. Participants were provided with a blank A4 sheet of paper for writing. Results were evaluated using a computer program we created, which assessed the positioning, initiation of writing, and text distribution on the page, including margins, line spacing, and word spacing. The shape, slant, and size of letters, as well as writing speed and spontaneity, were also analyzed.

**Result:**

Distinct handwriting features were identified that progressed in patients during the follow‐up graphological assessments conducted a year later. Patients experienced difficulties understanding the tasks of the graphological study. Notably, there was a lack of top and left margins, with large right margins. The right and left edges were uneven, and there were varying intervals between lines and words. Letters within words exhibited different sizes, with larger letters predominating, often expanded both vertically and horizontally, likely due to age‐related hyperopia. There were noticeable gaps between letters in words, and words were frequently left unfinished. Substitutions of letters and syllables within words were observed, along with simplified, angular letter forms. Lines were uneven and tended to rise, with variations in letter slant within a single word. The pressure applied during writing fluctuated continuously, resulting in intermittent writing, slow pace, and a lack of spontaneity. Overall, the handwriting was difficult to decipher.

**Conclusion:**

Our study has highlighted characteristic handwriting traits that progress in patients with Alzheimer's disease. Further annual examination of handwriting samples from these patients is necessary.

